# Burst Ultrafast Laser Welding of Quartz Glass

**DOI:** 10.3390/ma18051169

**Published:** 2025-03-06

**Authors:** Xianshi Jia, Yinzhi Fu, Kai Li, Chengaonan Wang, Zhou Li, Cong Wang, Ji’an Duan

**Affiliations:** 1 State Key Laboratory of Precision Manufacturing for Extreme Service Performance, College of Mechanical and Electrical Engineering, Central South University, Changsha 410083, China; 221026@csu.edu.cn (X.J.); f2254923917@163.com (Y.F.); likai01@csu.edu.cn (K.L.); wangcong@csu.edu.cn (C.W.); duanjian@csu.edu.cn (J.D.); 2 School of Urban Design, Wuhan University, Wuhan 430072, China; 3 School of Intelligent Manufacturing, Hunan First Normal University, Changsha 410221, China; lit_ww@hnfnu.edu.cn

**Keywords:** femtosecond laser, laser welding, quartz glass, shadow imaging

## Abstract

Ultrafast laser welding of transparent materials has been widely used in sensors, microfluidics, optics, etc. However, the existing ultrafast laser welding depths are limited by the short laser Rayleigh length, which makes it difficult to realize the joining of transparent materials in the millimeter depth range and becomes a new challenge. Based on temporal shaping, we realized Burst mode ultrafast laser output with different sub-pulse numbers and explored the effect of different Burst modes on the welding performance using high-speed shadow in situ imaging. The experimental results show that the Burst mode femtosecond laser (twelve sub-pulses with a total energy of 28.9 μJ) of 238 fs, 1035 nm and 1000 kHz can form a molten structure with a maximum depth of 5 mm inside the quartz, and the welding strength can be higher than 18.18 MPa. In this context, we analyzed the transient process of forming teardrop molten structures inside transparent materials using high-speed shadow in situ imaging detection and systematically analyzed the fracture behavior of the samples. In addition, we further reveal the Burst femtosecond laser welding mechanism of transparent materials comprehensively by exploring the difference in welding performance under the effect of Burst modes with different sub-pulse numbers. This paper is the first to realize molten structures in the range of up to 5 mm, which is expected to provide a new welding method for curved surfaces and large-size transparent materials, helping to improve the packaging strength of photoelectric devices and the window strength of aerospace materials.

## 1. Introductions

In recent times, the demand for transparent material joining techniques across diverse sectors has been steadily growing, and ultrafast laser manufacturing technology has garnered significant attention [1,2,3]. Precise and efficient welding of transparent materials is essential in numerous areas. For instance, in automotive part manufacturing, electronic product processing, optical device fabrication, medical instrument production, chip and device packaging in the microelectronics and optoelectronics domains, intelligent sensing device manufacturing, as well as the assembly of aerospace optical components [4].

Ultrafast laser welding technology, with its merits of high precision, high efficiency, and high environmental friendliness, offers strong support for the development of these fields [5]. Thus, it holds great practical significance and broad prospects. Ultrafast lasers can directly interact with transparent materials such as glass through nonlinear absorption processes, including multi-photon absorption, tunnel ionization, and avalanche ionization [6,7,8,9]. Additionally, it can be employed to locally melt the glass interface. When the laser focus is positioned at the interface between two adjacent glass pieces, the resolidification of the glass material results in bond formation. As a result, ultrafast lasers are playing an increasingly crucial role in the glass welding field [10,11,12].

Lately, glass laser welding, as a novel processing technology, has gradually emerged as a prominent research area in the materials processing field due to its distinct advantages. Yichen Huang et al. [13] used a high-repetition-rate infrared picosecond laser to weld non-optical-contact soda-lime glass. They systematically explored the impacts of parameters like laser power, number of scanning passes, scanning pitch, scanning direction, and focal position on the weld seam. The findings indicated that under optimal process parameters, an effective connection could be achieved when the defocus amount was within ±200 μm. The weld seam size reached 10 mm × 5 mm, and the welding strength was 24.85 MPa. Guodong Zhang et al. [14] introduced a spatial beam shaping approach to generate customized Bessel beam lasers for aluminosilicate glass welding and studied the influence of focal field modulation and heat accumulation effects. The research revealed that the ultrafast Bessel beam enhanced the focal tolerance of glass welding, which was three times that of ultrafast Gaussian beam welding, effectively improving the mechanical and optical quality of ultrafast laser welding.

Apart from changing laser parameters (laser power, repetition rate, etc.), an intermediate layer can also be added to glass to assist welding and improve welding strength. Hao Chen and his colleagues [15] made use of pressure and water as intermediate layers separately to enhance the femtosecond laser welding of non-optical-contact glass. The research outcomes demonstrated that water-assisted femtosecond laser welding outperformed other methods in terms of welding uniformity and quality. To efficiently achieve high-strength and transparent glass welded joints, Wang Hao et al. [16] inserted a 30 nm Ti coating as an absorption layer between two glass pieces and employed nanosecond pulse lasers to weld the titanium-coated glass. At the same time, Wang Cong et al. [17] utilized silver nanofilms to assist femtosecond lasers in achieving high-quality welding of quartz glass.

The above-mentioned studies mainly centered on exploring the impacts of laser parameters and intermediate layers on welding strength. However, the influence of the multi-parameter mode of pulse trains (Burst) on welding strength remains uncertain. Currently, some ultrafast lasers are equipped with the Burst operation mode [18,19]. Burst mode, also referred to as the pulse train mode, differs from the traditional mode where a single pulse is output per cycle. In Burst mode, several sub-pulses are grouped together, and a sequence of sub-pulses is output within each cycle, presenting as a pulse train in the entire time domain. As a result, Burst mode has an extremely high repetition rate within its sub-pulse train, characterized by strong pulse energy in a short time period and high ablation efficiency. This Burst mode is capable of altering the energy distribution and temporal characteristics of femtosecond lasers and has a notable impact on the material processing process.

In this regard, Théo Guilberteau et al. [20] investigated the elongation changes produced by femtosecond lasers with a Bessel beam shape in fused silica under three distinct working modes: the single-pulse, MHz-Burst, and GHz-Burst modes. Both the single-pulse and MHz-Burst modes can achieve modifications exceeding 900 µm, while the maximum modification achieved by the GHz-Burst mode is 500 µm. Miglė Mackevičiūtė et al. [21] also carried out a comparative study on the modification lengths and positions under the MHz- and GHz-Burst mechanisms. Compared with the MHz-Burst state, the modifications formed in fused silica under the GHz Burst are more uniform and have a longer lateral length. Under the polarization control mode, the longitudinal length reaches 1 mm and the lateral length reaches 32 µm. M A Murzakov et al. [22] studied the effects of different pulse numbers and different pulse modulation shapes on the formation process of modifications in transparent media and discovered that using Burst mode would result in an increase in the geometric parameters, namely the length and width, of the modified region. For a single pulse, the length of the modified region is 426 µm, while it rises to 579 µm in Burst mode.

In addition, Xie Xiaozhu et al. [23] put forward a material processing approach for cutting and drilling micro-holes with diverse shapes on fused silica by making use of the Burst mode of ultrafast lasers. Through ablation experiments, they delved into the processing mechanism between ultrafast lasers and fused silica. In contrast to the non-Burst mode, in Burst mode, the ablation diameter and the heat-affected zone (HAZ) were notably decreased, which is conducive to enhancing the quality of laser cutting and drilling. Kun He et al. [24] conducted research on the ablation of fused silica in the femtosecond laser Burst mode and normal pulse mode via a three-dimensional two-temperature model and validated the findings through experiments. They explored the incubation effect (pre-pulse effect) triggered by the sub-pulses in the Burst and unearthed an optimal combination of the sub-pulse number and the sub-pulse separation time. Under this combination, the maximum machining efficiency and the minimum thermal damage area can be attained.

Zhou Li et al. [25] employed a Burst mode femtosecond laser (featuring four sub-pulses and a total energy of 132.5 μJ) with parameters of 238 fs, 1035 nm, and 200 kHz to perform micro-welding on non-optical-contact sapphire/metal, yielding a smooth and uniform interface. The welding strength reached as high as 11 MPa. Against this backdrop, they systematically analyzed the welding performance and mechanism of ultrafast lasers in the Burst mode and the single-pulse mode. Haodong Ren et al. [26] presented a novel method for welding glass and metal using ultrafast pulse lasers in Burst mode. With the ultrafast lasers in Burst mode, the volume of the molten pool was significantly enlarged, and the height of the molten pool reached around 350 μm, which was over an order of magnitude greater than that of traditional ultrafast lasers (10–20 μm). It could complete the welding process of metal and solder as well as the fusion-welding process of solder and glass in one step, preventing the severe damage to glass and solder inflicted by the single-pulse mode.

Currently, most of the research focuses on the modification of transparent materials by Burst mode ultrafast lasers. However, the welding mechanism, process, and technology of welding transparent materials using Burst mode ultrafast lasers have not been systematically studied. In addition, focal sensitivity also deserves attention. At present, the highest is the teardrop structure with a length of 1 mm [20,21,22], and its depth is limited. The welding process with a higher focal range will broaden the welding window and have greater practicality. It is worth mentioning that high-speed imaging technology plays a crucial role in the research. However, the current research has only analyzed the formation process of the teardrop structure and has not been used to analyze the actual welding process. In this study, by using a high-speed camera combined with special filters, the dynamic evolution process of the molten pool at the moment of welding was captured, clearly presenting the flow direction, fluctuation frequency, and size change of the molten pool [27]. The resolution was as high as 1024 × 1024 pixels, and the frame rate reached 3000 frames per second, far exceeding the imaging accuracy of similar studies. Through these high-definition and real-time image data, the stability of the welding process can be accurately analyzed, providing an intuitive basis for optimizing welding parameters and greatly promoting the welding technology of transparent materials to a new level.

Based on the above issues, this paper established a femtosecond laser welding experimental system and conducted an experiment on the induced modification of quartz glass by femtosecond lasers in Burst mode. The influence laws of different numbers (one, two, four, six and twelve) of sub-pulses on the teardrop-like molten structures in quartz glass were systematically analyzed using the method of controlling variables. Subsequently, the dynamic process of quartz glass under femtosecond lasers in Burst mode was photographed using a shadow imaging system, and the differences in the different numbers of sub-pulses were analyzed. Finally, welding experiments on quartz glass with different numbers of sub-pulses of femtosecond lasers in Burst mode were carried out, and the variation of the welding strength with the defocus amount was explored.

## 2. Materials and Methods

### 2.1. Equipment and Materials

**Figure 1:**
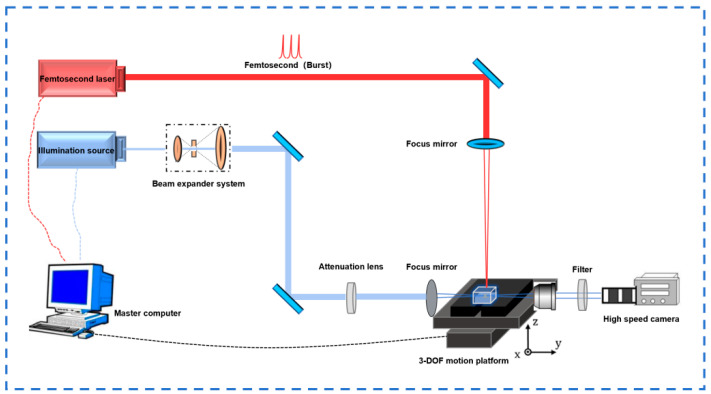
A schematic diagram of the femtosecond laser light path.

The laser welding system is shown in Figure 1. The femtosecond laser beam is emitted from the output port of the femtosecond laser (HR-Platform-0203, Wuhan Huaray Precision Laser Co., Ltd., Wuhan, China). After passing through three high-reflectivity mirrors, it enters the galvanometer system. The galvanometer is installed together with the focusing lens (focal length (f = 80 mm)) on the lifting platform. The rotation of the galvanometer is controlled by an internal motor to achieve the scanning function. Also, considering the convenience of cleaning the internal lenses of the galvanometer, a dust-proof device is installed in front of the galvanometer entrance. After being adjusted by the galvanometer and passing through the focusing lens, the laser beam is finally focused on the surface of the quartz glass sample on the stage for welding. Both the femtosecond laser and the galvanometer system can be controlled by the main control computer. The laser parameters are shown in Table 1.

The high-speed imaging system is shown in Figure 1. The femtosecond laser beam is also emitted from the output port of the femtosecond laser. After passing through three high-reflectivity mirrors, the optical path is lifted. Then, it passes through the focusing lens and is focused on the surface of the quartz glass sample on the three-degree-of-freedom motion platform. At the same time, the illumination beam is emitted from the output port of the illumination source. First, it passes through the beam expander, then the optical path is lifted to the same height and irradiates the sample surface. Subsequently, it passes through the attenuator mirror group, the focusing lens and the filter in sequence, and finally serves as the background light of the imaging system and is captured by the high-speed camera. The high-speed camera shadow imaging system is used to capture the dynamic interaction process between the femtosecond laser and the quartz glass, providing an intuitive basis for optimizing the welding parameters (the number of sub-pulses, defocus amount, etc.).

**Figure 2:**
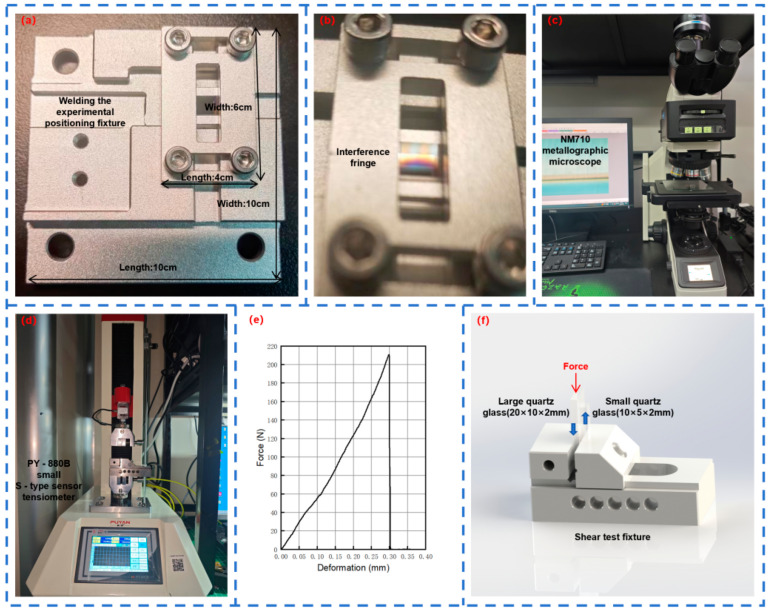
(**a**) Top view of the installation of the welding fixture; (**b**) interference fringes generated by the installation; (**c**) NM710 metallographic microscope; (**d**) small S-type sensor tension meter; (**e**) stress–strain state diagram; (**f**) schematic diagram of the application of shear test load.

Quartz glass is a colorless and transparent inorganic amorphous material. It has high transparency, being able to transmit approximately 90% of visible light and almost all ultraviolet light. The experimental material for characterization is fully polished quartz glass with dimensions of 15 × 15 × 5 mm. The six-sided polishing is carried out to facilitate the observation of the microscopic morphology of the molten structure. The experimental materials for welding are double-polished quartz glass with dimensions of 20 × 10 × 2 mm and double-polished quartz glass with dimensions of 10 × 5 × 2 mm. Double-sided polishing can eliminate surface microscopic defects and non-uniformity, thereby improving the quality and reliability of the welded joints. Before the welding experiment, the quartz glass is fixed using a positioning fixture, and appropriate pressure is applied through adjusting bolts. The interference fringes are observed to control the gap between the two pieces of quartz glass to meet the optical contact condition, as shown in Figure 2a,b.

### 2.2. Experiments and Characterization

To observe the teardrop-shaped molten structure generated when the laser acts on the quartz glass and the cross-sectional view of the quartz glass in the welding experiment, the NM710 metallographic microscope (Nexcope, Ningbo Yongxin Optics Co., Ltd., Ningbo, China) with magnification and measurement functions is an indispensable tool in this experiment, as shown in Figure 2c. The wide-field adjustable eyepiece provides a 10-fold magnification, and the infinity-corrected flat-field objectives offer magnifications of 5-fold, 10-fold, 20-fold, 50-fold, and 100-fold, respectively.

To determine the strength of the welded joint, a PY-880B small S-type sensor tensiometer is used for the shear test, as shown in Figure 2d. To ensure a uniform load distribution, the small piece of quartz glass is placed on a fixed platform, while the large piece of quartz glass passes through the gap of the fixed platform. A load is gradually applied to it until the two pieces of quartz glass separate. The stress–strain state diagram is shown in Figure 2e and the specific scheme is shown in Figure 2f.

## 3. Results and Discussion

### 3.1. Femtosecond Laser Melting Characteristics of Quartz Glass in Burst Mode

**Figure 3:**
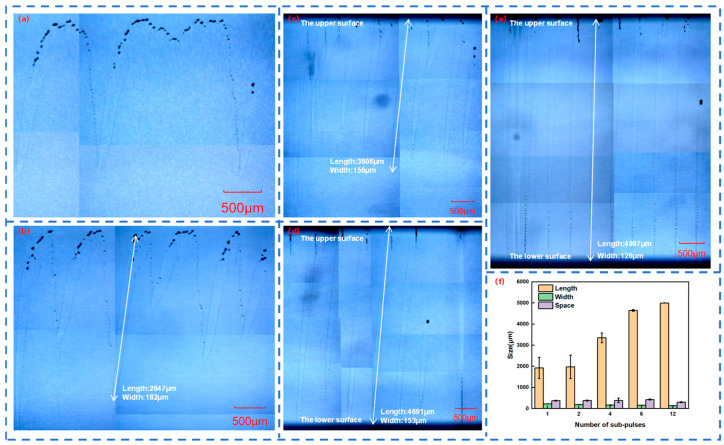
Molten structure in a quartz glass block with: (**a**) 1 sub-pulse; (**b**) 2 sub-pulses; (**c**) 4 sub-pulses; (**d**) 6 sub-pulses; and (**e**) 12 sub-pulses in Burst mode; (**f**) length, width and spacing of the molten structures of quartz glass with different numbers of sub-pulses in Burst mode.

When femtosecond laser acts on quartz glass, a teardrop-shaped molten structure is generated inside the quartz glass. By adjusting the number of sub-pulses in Burst mode, the instantaneous energy density and heat conduction process generated by the femtosecond laser in the quartz glass can be controlled. Therefore, different Burst modes will lead to different melting depths and thus form different teardrop-shaped molten structures. Femtosecond lasers with different sub-pulses in Burst mode were respectively selected to act on the quartz glass, and the length, width, and spacing of the teardrop-shaped molten structures were measured, as shown in Figure 3a–e.

Figure 3f shows the dependence of the length, width, and spacing of the teardrop-shaped molten structures in quartz glass on the number of sub-pulses in Burst mode. As the number of sub-pulses increases, the length of the molten structure gradually increases, the width gradually decreases, and the spacing gradually increases. However, when the number of sub-pulses is 12, the length of the molten structure reaches the thickness limit of the quartz glass, which is 5 mm, and its spacing also decreases to some extent, forming slender and uniform teardrops.

In addition, an increase in the number of sub-pulses leads to a corresponding decrease in the energy of each sub-pulse when the total energy remains unchanged. When the number of sub-pulses of the femtosecond laser increases from 1 to 12, the width of the molten structure decreases from 221 μm to 132 μm. This explains why the width of the teardrop-shaped molten structure decreases with the increase in the number of sub-pulses. However, an increase in the number of sub-pulses means that several sub-pulses act on the quartz glass successively. After one sub-pulse forms a plasma channel, the next sub-pulse expands the plasma channel on this basis, repeating this process until the action of the last sub-pulse ends. When the number of sub-pulses of the femtosecond laser increases from 1 to 12, the length of the molten structure increases from 1923 μm to 4993 μm. This explains why the length of the teardrop-shaped molten structure increases with the increase in the number of sub-pulses.

### 3.2. Dynamic Process of Femtosecond Laser Processing of Quartz Glass Under Burst Mode

**Figure 4:**
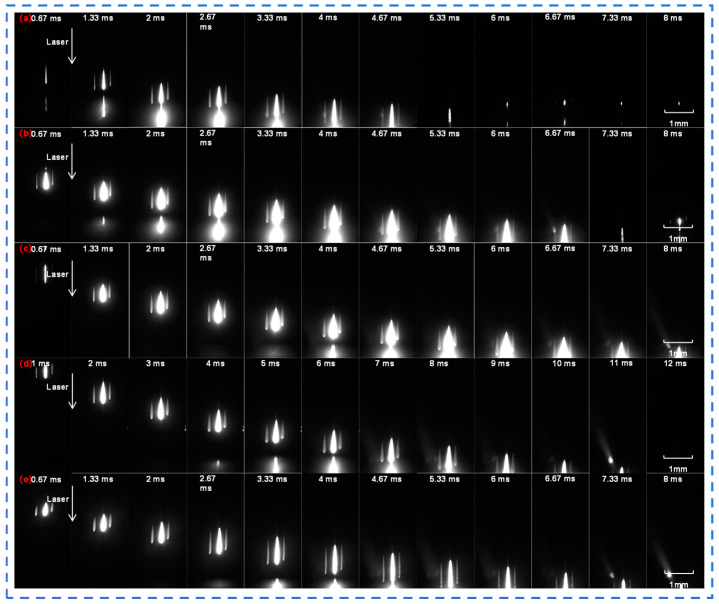
High-speed imaging of femtosecond laser acting on quartz glass with different numbers of sub-pulses in Burst mode (without background light): (**a**) 1; (**b**) 2; (**c**) 4; (**d**) 6; and (**e**) 12.

**Figure 5:**
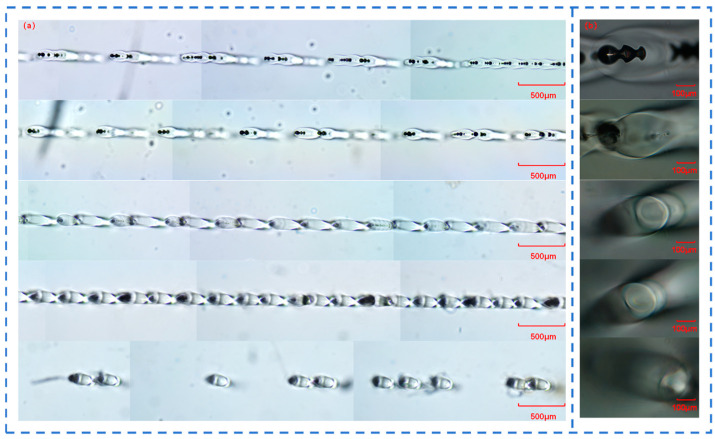
Top view of femtosecond laser welding of quartz glass with different numbers of sub-pulses in Burst mode: (**a**) 5-times magnification; (**b**) 20-times magnification. From top to bottom the number of sub-pulses are 1, 2, 4, 6, 12.

In order to observe the process of femtosecond laser acting on quartz glass intuitively and clearly, a high-speed camera is used to capture the action process. Figure 4 and Figure 5 are the photos of the femtosecond laser acting on quartz glass taken by the camera when the number of sub-pulses is one, two, four, six, and twelve, respectively, and the time interval between each photo is 0.67 ms (1 ms when there are six sub-pulses). There was no background light when Figure 4 was taken.

When the number of sub-pulses is 1, a single laser action trace is visible in the photograph. At this point, the femtosecond laser interacts with the quartz glass in the form of a single pulse. The material undergoes relatively simple and straightforward changes, with only minor local alterations occurring at the laser-action point, such as electron excitation and slight lattice perturbation. Since there is no superposition effect from subsequent sub-pulses, energy deposition is relatively uncomplicated.

When the number of sub-pulses is two, compared to the case of one sub-pulse, the trace exhibits certain modifications. The time interval between the two sub-pulses allows the second sub-pulse to act once more on the material area that has already been altered by the first sub-pulse. This results in more intricate energy deposition within the quartz glass, more prominent changes in the action area, and the degree of quartz glass modification starts to intensify.

When the number of sub-pulses reaches four, six, and twelve, it can be observed that the action traces gradually expand, and the action process becomes increasingly complex. As the number of sub-pulses rises, multiple sub-pulses act on the quartz glass successively at an interval of 22 ns. Energy accumulates continuously within the material, rendering the physical and chemical changes in the action area more substantial. Physical changes include material stress and structural alterations, and chemical changes include the breakage and recombination of chemical bonds [28]. Moreover, the shape and size of the action area display distinct characteristics compared to when there are fewer sub-pulses.

### 3.3. Femtosecond Laser Welding Characteristics of Quartz Glass in Burst Mode

**Figure 6:**
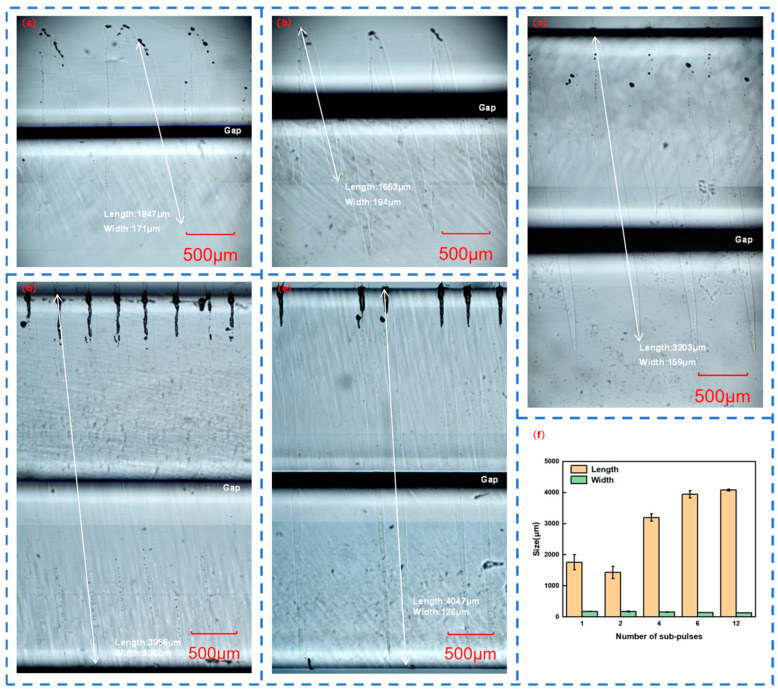
Molten structure of the welding of two quartz glass pieces with: (**a**) 1 sub-pulse; (**b**) 2 sub-pulses; (**c**) 4 sub-pulses; (**d**) 6 sub-pulses; and (**e**) 12 sub-pulses in Burst mode; (**f**) length and width of the molten structures of quartz glass with different numbers of sub-pulses in Burst mode.

In order to compare the similarities and differences between the molten structures in the welding process and those in the characterization process, femtosecond lasers with different numbers of sub-pulses in Burst mode were used to weld two pieces of six-sided polished quartz glass. The top view of the welded quartz glass was observed under a microscope, as shown in Figure 6, where Figure 5a is magnified 50 times and Figure 5b is magnified by 200 times. The cross-sectional views are shown in Figure 6a–e. The welding experimental conditions were determined to meet the optical contact conditions by observing the interference fringes between two fused silica sheets. Meanwhile, the length and width of the teardrop-shaped molten structures were measured, as shown in Figure 6f.

Figure 6f shows the dependence of the length and width of the teardrop-shaped molten structures of the quartz glass during the welding process on the number of sub-pulses in Burst mode. As the number of sub-pulses increases, the length of the molten structure also gradually increases, and the width also gradually decreases. However, when the number of sub-pulses is twelve, the length of the molten structure reaches the total thickness limit of the two pieces of quartz glass, which is 4 mm, and also forms slender and uniform teardrops.

Different from the molten structures in the characterization process, the length and width of the molten structures in the welding process are reduced to different extents. When the number of sub-pulses is one, the number of molten structures with smaller volumes is significantly reduced; when the number of sub-pulses is two, some molten structures do not penetrate through the two pieces of quartz glass; when the number of sub-pulses is four, the long and short molten structures are arranged alternately; when the number of sub-pulses is six, the molten structures are relatively neat and uniform; when the number of sub-pulses is twelve, the number of molten structures decreases and the distribution is uneven.

**Figure 7:**
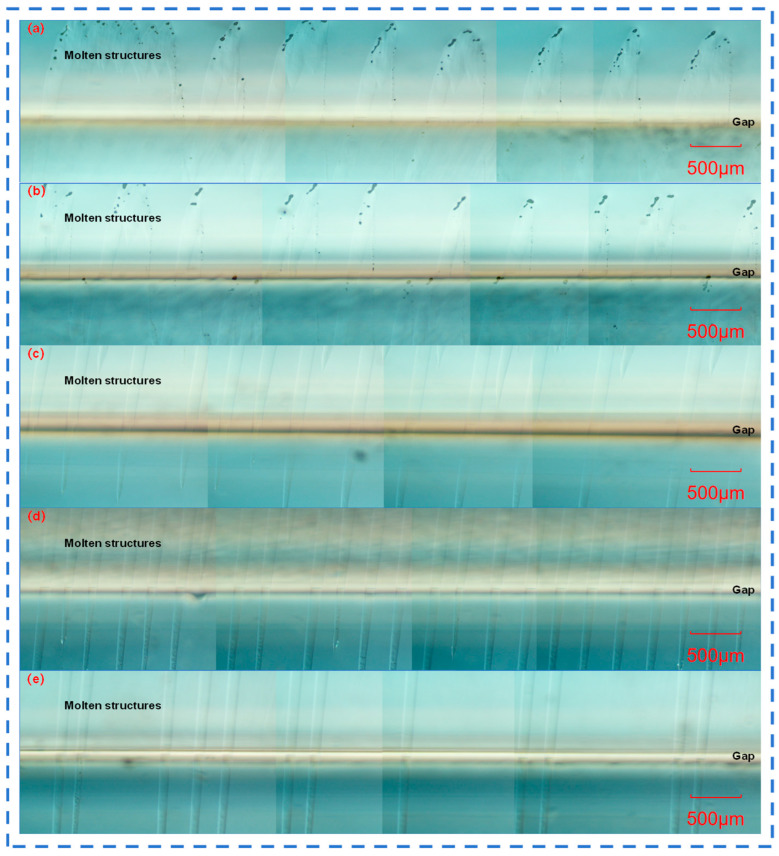
Cross-sectional view of femtosecond laser welding of two quartz glass pieces with different numbers of sub-pulses in Burst mode: (**a**) 1; (**b**) 2; (**c**) 4; (**d**) 6; (**e**) 12.

Based on the observation of the gap morphology of the quartz glass at different sub-pulses in the Burst mode in Figure 7, the reasons for the above differences are as follows: The characterization material is a quartz glass block, and the femtosecond laser in Burst mode acts on it to form molten structures with regular arrangement; while the welding material is two pieces of quartz glass stacked, with a gap in between. When the femtosecond laser in Burst mode is focused on the lower quartz glass, high-temperature plasma and high-density plasma are generated. The plasma channel expands upward, and when it reaches the gap, part of the energy will be lost for the efficient mutual fusion and connection of the quartz glass, and the remaining energy will cross the gap and continue to expand the plasma channel upward.

### 3.4. Analysis of Femtosecond Laser Welding Strength in Burst Mode

#### 3.4.1. Variation of Quartz Glass Welding Strength with the Number of Bursts

The Burst mode of the femtosecond laser can change the energy distribution and time characteristics of the femtosecond laser. To study the influence of the number of Burst sub-pulses of different femtosecond lasers on the welding strength of quartz glass, experiments were conducted by selecting one, two, four, six, and twelve sub-pulse numbers. To ensure the accuracy of the experiment, the average value was obtained after four experiments for each group of parameters. Due to Burst mode causing the laser energy to be redistributed and the focal position to be changed again, different defocusing amounts (z-values) were selected for different sub-pulse numbers.

**Figure 8:**
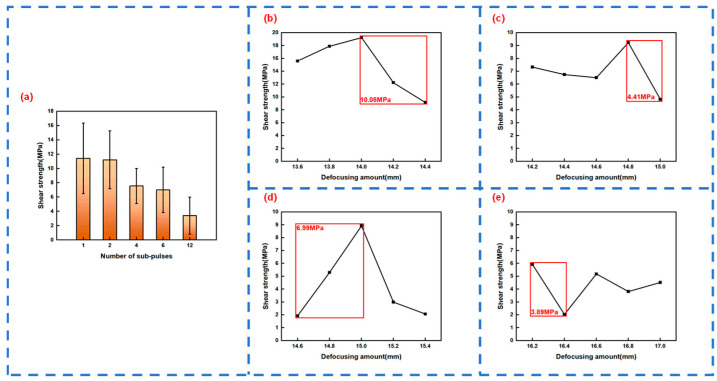
(**a**) Welding strength of quartz glass as a function of the number of Burst sub-pulses. Line chart of the welding strength of quartz glass as a function of the defocusing amount z-value when the number of sub-pulses is (**b**) 2; (**c**) 4; (**d**) 6; and (**e**) 12.

Figure 8a shows the change in the welding strength of quartz glass with the number of Burst sub-pulses at a repetition rate of 1000 kHz and a single-pulse energy of 28.9 μJ. It can be seen from Figure 8a that as the number of Burst sub-pulses increases, the width of the molten structure decreases, the area of the acting area decreases, and the welding strength of quartz glass gradually decreases. Among them, the average welding strength when the number of sub-pulses is one is 11.425 MPa, and the average welding strength when the number of sub-pulses is two is 11.2125 MPa, with little difference; the average welding strength when the number of sub-pulses is four is 7.565 MPa, and the average welding strength when the number of sub-pulses is six is 7.01 MPa, also with little difference; the average welding strength when the number of sub-pulses is twelve is 3.415 MPa.

In addition, the maximum welding strength of these five groups of experiments also gradually decreases with the increase in the number of Burst sub-pulses, which are 18.18 MPa, 16.63 MPa, 11.11 MPa, 9.76 MPa, and 7.28 MPa in sequence. As the number of sub-pulses increases, the width of the teardrop-shaped molten structure decreases, the spacing increases and the area of the modified region decreases, thus the welding strength decreases accordingly.

#### 3.4.2. Variation of Quartz Glass Welding Strength with the Defocusing Amount z-Value

In order to study the influence of different defocusing amounts z-values on the welding strength of quartz glass, different defocusing amounts z-values were selected for experiments when the number of sub-pulses was two, four, six, and twelve, respectively. By comparing the four line graphs in Figure 8b–e, it can be seen that with the increase in the number of sub-pulses, the welding strength gradually decreases, which is consistent with the experimental results of three. By observing each line graph, it can be found that within the allowable error range, as the defocusing amount z-value increases, the welding strength generally shows a trend of first increasing and then decreasing, indicating that when the laser focus is located slightly below the center of the two pieces of quartz glass, the welding strength is the largest.

In addition, under the same defocusing amount z-value interval (0.8 mm), the variation ranges of the welding strength are 10.08 MPa, 4.41 MPa, 6.99 MPa, and 3.89 MPa, respectively. It can be found that within the allowable error range, the variation range of the welding strength decreases with the increase in the number of sub-pulses, that is, the more the number of sub-pulses, the smaller the variation range of the welding strength, and the higher the stability of quartz glass welding. This is because as the number of sub-pulses increases, the length of the teardrop-shaped molten structure increases, and the length and uniformity of the modified region increase. This Burst mode is extremely suitable for micro-curved surface welding.

### 3.5. Fracture Interface of Welded Quartz Glass in Burst Mode

**Figure 9:**
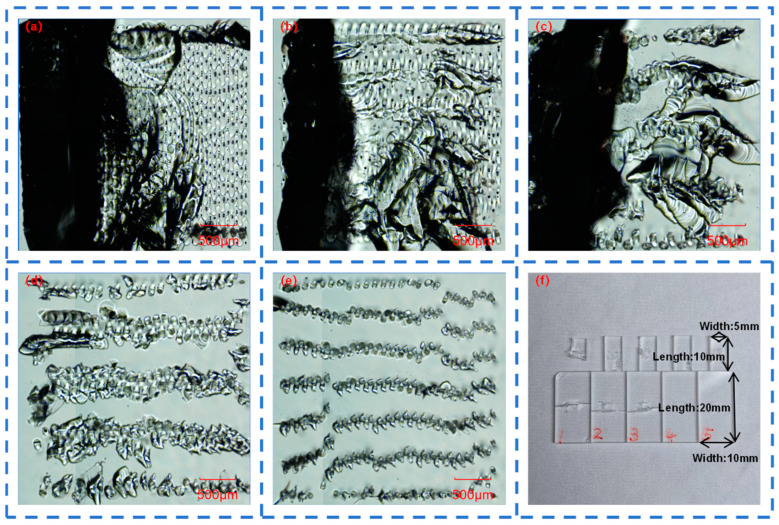
Morphology of the fracture interface of quartz glass when the number of sub-pulses is: (**a**) 1; (**b**) 2; (**c**) 4; (**d**) 6; and (**e**) 12; (**f**) fracture conditions of quartz glass.

After the welding strength test, the fracture interface of the quartz glass was observed using a microscope. One set of experimental data was selected for analysis, as shown in Figure 9a–e. When the number of sub-pulses is one, the molten structures on the quartz interface are dense but not sufficiently molten. When the number of sub-pulses is two, the molten structures on the quartz interface are still dense and sufficiently molten, and there is a residual molten sample on the right side of the large piece of quartz glass. When the number of sub-pulses is four, the number of molten structures on the quartz interface decreases but they are sufficiently molten. When the number of sub-pulses is six, two adjacent rows of molten structures on the quartz interface merge into one row. When the number of sub-pulses is twelve, the row spacing of the molten structures on the quartz interface is large and the melting is not sufficient.

When the number of sub-pulses in Burst mode is one, two, and four, the corresponding welding strengths are 9.21 MPa, 11.97 MPa, and 11.11 MPa, respectively. In these three cases, the large piece of quartz glass fractures, and in the first case, the small piece of quartz glass also shatters. When the number of sub-pulses in Burst mode is six and twelve, the corresponding welding strengths are 7.21 MPa and 7.28 MPa, respectively, and the quartz glass pieces are generally intact. As shown in Figure 9f, the morphology of the fracture section thus fully corroborates the magnitude of the welding strength.

## 4. Conclusions

In this paper, taking the typical transparent brittle hard material, quartz glass, as the welding material, combined with the morphological distribution of teardrop-shaped molten structures in the quartz block and the dynamic process of the interaction between the laser and quartz during shadow imaging, the difference in the welding strength of quartz glass under femtosecond lasers with different Burst parameters is explored. The main conclusions are as follows:

(1) With the increase in the number of sub-pulses, the length of the molten structure gradually increases, while the width gradually decreases. When the number of sub-pulses is twelve, the average length of the molten structure exceeds 5 mm, the average width is 132 μm, and the average spacing is 300 μm.
(2) The dynamic process of femtosecond laser acting on quartz glass was observed by using plasma imaging technology. With a resolution as high as 1024 × 1024 pixels and a frame rate reaching 3000 frames per second, it can accurately analyze the stability of the welding process and provide an intuitive basis for optimizing the welding parameters.
(3) When using the Burst mode, the pulse width of the femtosecond laser is 238 fs, the wavelength is 1035 nm, and the repetition frequency is 1000 kHz (one sub-pulse and the total energy is 28.9 μJ). When the z-value is 14 mm, the maximum welding strength of the quartz glass can reach 18.18 MPa. The maximum welding strengths of the other four groups of experiments are 16.63 MPa, 11.11 MPa, 9.76 MPa, and 7.28 MPa, respectively.
(4) With the increase in the number of sub-pulses, the area of the modified region decreases, and the average welding strength of the quartz glass gradually decreases from 11.425 MPa to 3.415 MPa. When the number of sub-pulses is determined, with the increase of the defocusing amount z-value, the welding strength generally shows a trend of first increasing and then decreasing. The morphology of the fractured part fully confirms the magnitude of the welding strength.

When the number of sub-pulses is relatively large, the width of the molten structures in quartz glass narrows, and the quantity of these molten structures decreases, which restricts the strength. In the future, it is expected that the welding strength could be enhanced by reducing the scanning spacing or increasing the number of scanning times.

## Figures and Tables

**Table 1:** Laser parameters.

Parameter	Value
Central wavelength	1035 nm
Focused spot diameter	33.245 μm
Repetition rate	1000 kHz
Pulse width	200 fs
Burst total energy	28.9 μJ
Number of sub-pulses	1/2/4/6/12
Sub-pulse interval time	22 ns
Processing speed	20 mm/s
Processing area	3 × 3 mm^2^

## Data Availability

The original contributions presented in this study are included in the article. Further inquiries can be directed to the corresponding author.
